# Supplementary material for: Bioinformatics Prediction and Experimental Verification Identify CAB39L as a Diagnostic and Prognostic Biomarker of Kidney Renal Clear Cell Carcinoma
Source: Medicina (Kaunas). 2023 Apr 6;59(4):716. doi: 10.3390/medicina59040716 (PMC10145756; doi:10.3390/medicina59040716)
Supplement: Supplementary file 1 [file medicina-59-00716-s001.zip › medicina-2241596-supplementary.pdf]

Table S1. Top 15 upregulated and downregulated DEGs.

Gene_name	log_2FoldChange	padj
HEPACAM2	5.817395203	$1.55372*10^{-84}$
AQP6	5.132763465	$2.79194*10^{-89}$
ATP6V0A4	5.130930949	$9.75504*10^{-89}$
ATP6V1G3	5.08983415	$3.84498*10^{-22}$
CLDN8	5.085752819	$2.59077*10^{-39}$
TMEM213	4.712693704	$1.741*10^{-70}$
KLK1	4.625309281	$1.18356*10^{-60}$
RHCG	4.610154628	$2.63941*10^{-58}$
NMRK2	4.513983911	$2.74403*10^{-52}$
DMRT2	4.405467322	$3.48914*10^{-51}$
KLK15	4.340718871	$4.72159*10^{-21}$
NR0B2	4.31975483	$8.52054*10^{-44}$
CASP14	4.253412536	$2.06044*10^{-21}$
GPRC6A	3.954480516	$2.81457*10^{-15}$
ATP6V0D2	3.832991241	$3.4664*10^{-56}$
PAEP	−4.974078494	$4.29293*10^{-64}$
SAA1	−4.250709694	$1.6993*10^{-56}$
FDCSP	−3.922321566	$1.5007*10^{-28}$
SAA2-SAA4	−3.914853884	$1.59849*10^{-28}$
HP	−3.871135561	$4.27441*10^{-49}$
RTL1	−3.694637999	$4.66976*10^{-25}$
IGFBP1	−3.608571729	$9.3749*10^{-48}$
SAA2	−3.598976631	$5.36706*10^{-41}$
GOLGA6L7	−3.560697007	$4.07564*10^{-53}$
GOLGA6L2	−3.502127767	$1.95147*10^{-32}$
APOC3	−3.472616871	$4.71656*10^{-33}$
NEUROD4	−3.388766484	$4.89126*10^{-17}$
WFDC5	−3.246411154	$3.64906*10^{-21}$
SAA4	−3.218392443	$1.7035*10^{-32}$
TNNT1	−3.108489777	$5.46186*10^{-46}$
